# Osteoprotective Effects of Loganic Acid on Osteoblastic and Osteoclastic Cells and Osteoporosis-Induced Mice

**DOI:** 10.3390/ijms22010233

**Published:** 2020-12-28

**Authors:** Eunkuk Park, Chang Gun Lee, Eunguk Lim, Seokjin Hwang, Seung Hee Yun, Jeonghyun Kim, Hyesoo Jeong, Yoonjoong Yong, Seong-Hoon Yun, Chun Whan Choi, Hyun-Seok Jin, Seon-Yong Jeong

**Affiliations:** 1 Department of Medical Genetics, Ajou University School of Medicine, Suwon 16499, Korea; jude0815@hotmail.com (E.P.); dangsunsang@naver.com (C.G.L.); eunguk@ajou.ac.kr (E.L.); tjrwlshh@naver.com (S.H.); yun41101@ajou.ac.kr (S.H.Y.); danbi37kjh@hanmail.net (J.K.); 2 Department of Biomedical Sciences, Ajou University Graduate School of Medicine, Suwon 16499, Korea; 3 Nine B Company, Daejeon 34121, Korea; jhyesoo921@gmail.com (H.J.); yoonjoong9b@gmail.com (Y.Y.); tpohot10@nate.com (S.-H.Y.); 4 Natural Products Research Institute, Gyeonggi Institute of Science & Technology Promotion, Suwon 16229, Korea; cwchoi78@gmail.com; 5 Department of Biomedical Laboratory Science, College of Life and Health Sciences, Hoseo University, Asan 31499, Korea

**Keywords:** osteoporosis, natural plant, loganic acid, osteoblast, osteoclast, bone mineral density, ovariectomized mice

## Abstract

Osteoporosis is a common disease caused by an imbalance of processes between bone resorption by osteoclasts and bone formation by osteoblasts in postmenopausal women. The roots of *Gentiana lutea* L. (GL) are reported to have beneficial effects on various human diseases related to liver functions and gastrointestinal motility, as well as on arthritis. Here, we fractionated and isolated bioactive constituent(s) responsible for anti-osteoporotic effects of GL root extract. A single phytochemical compound, loganic acid, was identified as a candidate osteoprotective agent. Its anti-osteoporotic effects were examined in vitro and in vivo. Treatment with loganic acid significantly increased osteoblastic differentiation in preosteoblast MC3T3-E1 cells by promoting alkaline phosphatase activity and increasing mRNA expression levels of bone metabolic markers such as *Alpl*, *Bglap*, and *Sp7*. However, loganic acid inhibited osteoclast differentiation of primary-cultured monocytes derived from mouse bone marrow. For in vivo experiments, the effect of loganic acid on ovariectomized (OVX) mice was examined for 12 weeks. Loganic acid prevented OVX-induced bone mineral density loss and improved bone structural properties in osteoporotic model mice. These results suggest that loganic acid may be a potential therapeutic candidate for treatment of osteoporosis.

## 1. Introduction

Osteoporosis is a progressive skeletal disorder characterized by dysregulation of bone remodeling, resulting in systemic reduction of bone mass and a high risk of bone fracture [1]. In elderly or postmenopausal women, osteoporosis occurs frequently because of estrogen deficiency, which plays a critical role in bone homeostasis [2,3]. Deficiency of female hormones trigger an abnormal bone remodeling process between bone formation (osteoblasts) and resorption (osteoclasts) [4]. Osteoblasts are derived from mesenchymal stem cells and play a central role in the regulation of bone mineralization and formation [5,6]. Osteoclasts are members of the monocyte/macrophage lineage and are found on old bone surfaces that break down old or damaged bone cells [7]. Impaired regulation between bone formation and resorption leads to inappropriate bone remodeling processes with serious bone loss, resulting in osteopenia and osteoporosis [8,9]. Currently, pharmacological treatment of osteoporosis, especially in women during menopausal period, is focused on selective estrogen receptor modulators, bone resorption inhibitors, or stimulators of bone formation [10]. Long-term treatment is required for osteoporosis; however, some pharmacological medications still have limitations such as side effects and unmet needs [11,12,13]. Natural herbal plants have been widely used as modern alternative medicines for the treatment of various diseases due to fewer side effects and are appropriate for long-term use and multifactorial effects [14,15,16]. Specific single physiological compound(s) isolated from natural plants have therapeutic effects on various diseases such as inflammation, type 2 diabetes, and cancer [17,18,19]. *Gentiana* is a genus of flowering plants used as a traditional medicine for the treatment of inflammation, skin diseases, and fever [20,21]. The *Gentiana lutea* L. (GL) root has been reported to improve gastrointestinal motility and liver function [22,23,24]. In recent years, bioactive phytochemicals of GL, including iridoids (loganic acid), secoiridoids (gentiopicroside, sweroside, swertiamarin, and amarogentin), and xanthones (isogentisin) have been identified [25]. One of the bioactive compounds, gentiopicroside, promoted protective effects against bone-related diseases such as arthritis and osteoporosis in ovariectomized (OVX) mice [26,27]. However, the effects of other bioactive compound(s) isolated from GL have not yet been studied.

In this study, we aimed to identify bioactive constituent(s) responsible for the anti-osteoporotic effect of GL extract. We purified a phytochemical single compound, loganic acid, from the root extract of GL. In addition, we evaluated the effects of loganic acid on osteoblastic and osteoclastic cell differentiation and in an animal model with OVX-induced bone loss.

## 2. Results 

### 2.1. A Bioactive Component for Promoting Osteoblast Differentiation Isolated from GL Root Extract

**Figure 1:**
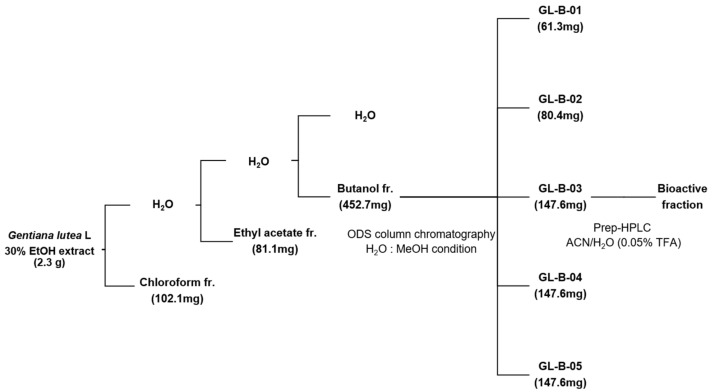
Extraction and purification of loganic acid from *Gentiana lutea* L. (GL). Ethanol extract of GL (30%) was fractionated using chloroform, ethyl acetate, and butanol. The butanol fraction was further fractionated into five sub-fractions by octadecylsilyl (ODS) gel column chromatography and eluted with acetonitrile (ACN) solution containing 0.05% trifluoroacetic acid (TFA) in a step-gradient manner (10 to 40%). Finally, a single bioactive compound was obtained. fr: fraction, HPLC: high-performance liquid chromatography.

To examine osteoprotective effects of *Gentiana lutea* L. (GL) root extract, we investigated alkaline phosphatase (ALP) activity in osteoblast cells and tartrate-resistant acid phosphatase (TRAP) activity in osteoclast cells. The results indicated that a 30% ethanol extract of GL root promoted ALP activity in MC3T3-E1 preosteoblast cells, whereas it inhibited TRAP activity in primary-cultured osteoclast cells (Supplementary Figure S1). A previous study has demonstrated that the GL root extract contains numerous bioactive components [28]. We screened bioactive component(s) isolated from GL extract for anti-osteoporotic agents. Briefly, an extract prepared from roots of GL was fractionated using chloroform, ethyl acetate, and butanol solvents (Figure 1).

**Figure 2:**
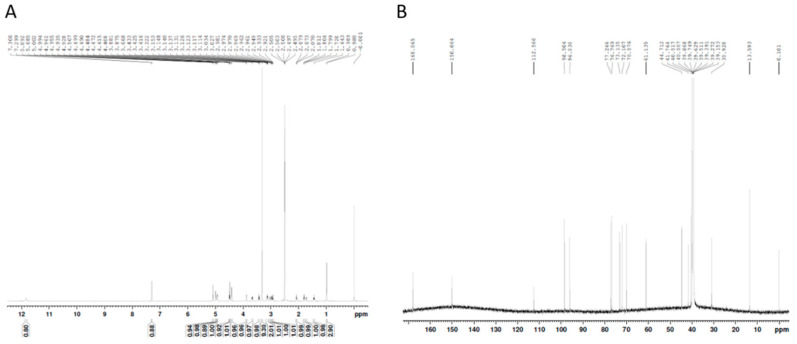
(**A**) Proton nuclear magnetic resonance (^1^H-NMR), (**B**) Carbon-13 nuclear magnetic resonance (^13^C-NMR) analysis of GL-B-03 fraction.

Next, each fraction was assessed for (ALP) activity, which is a characteristic marker of bone metabolism for enhancing bone formation [29]. As significantly increased ALP activity was observed in the butanol fraction, we further fractionated the butanol fraction into five sub-fractions (GL-B-01 to GL-B-05) by column chromatography. GL-B-03 showed the most significant effect on osteoblastic activity of MC3T3-E1 cells. The constituents of the final purified bioactive fraction (GL-B-03) were analyzed by proton nuclear magnetic resonance (^1^H-NMR) and carbon-13 nuclear magnetic resonance (^13^C-NMR) (Figure 2).

**Figure 3:**
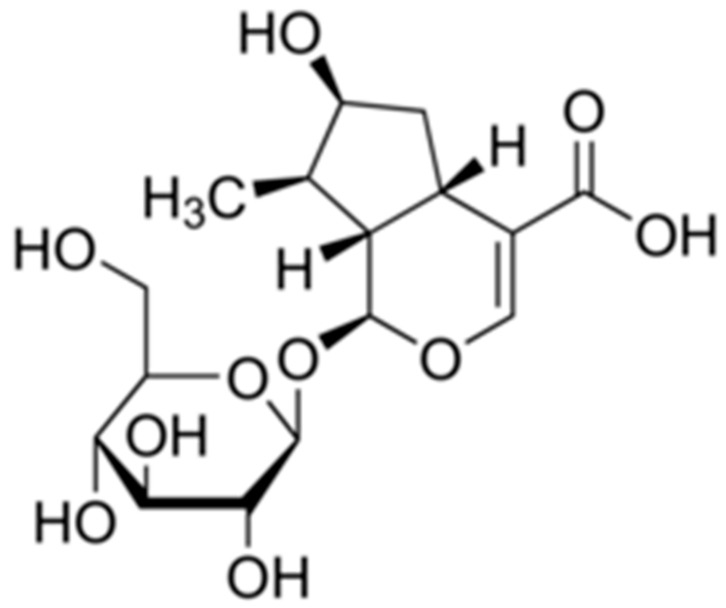
Molecular structure of loganic acid.

A single bioactive compound, loganic acid, (1S,4aS,6S,7R,7aS)-1-(β-D-Glucopyranosyloxy)-6-hydroxy-7-methyl-1,4a,5,6,7,7a-hexahydrocyclopenta[c]pyran-4-carboxylic acid, was identified (Figure 3), the molecular formula of which is C_16_H_24_O_10_.

### 2.2. Loganic Acid Promoted Osteoblast Differentiation in Preosteoblastic MC3T3-E1 Cells

**Figure 4:**
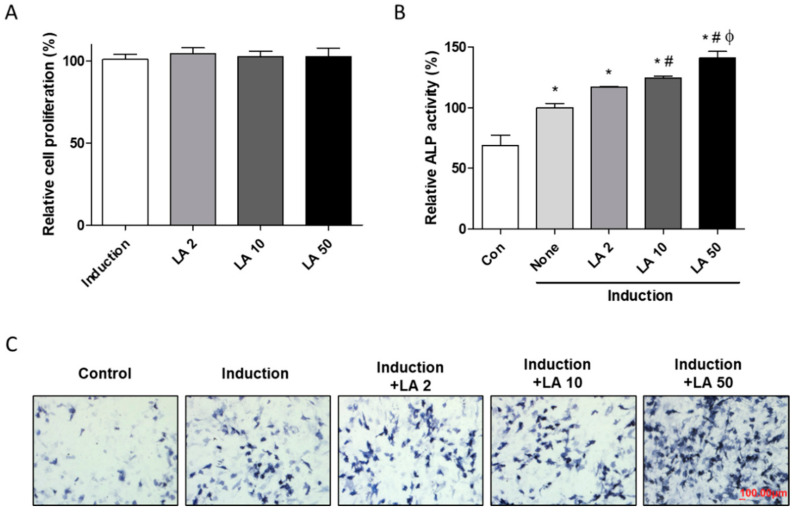
Effect of loganic acid on osteoblast differentiation in preosteoblastic MC3T3-E1 cells. Cells were incubated with osteoblast induction media (50 μg/mL of ascorbic acid and 10 mM of β-glycerophosphate) and co-cultured with loganic acid (2, 10, and 50 μM) for 3 days. Osteoblastic differentiation was assessed by (**A**) cell proliferation, ALP activity (**B**), and ALP staining (**C**). LA: loganic acid, Control: non-osteoblast induction media-treated group, None (Induction): osteoblast induction media-treated group. * *p* < 0.05 vs. Con, ^#^ *p* < 0.05 vs. None (Induction), ^ϕ^ *p* < 0.05 vs. LA2 (ANOVA with Tukey’s honest significant difference post hoc test).

To confirm the effects of the purified bioactive compound, identified as loganic acid, isolated from the GL extract, on bone formation in vitro, we evaluated osteoblast differentiation in MC3T3-E1 cells. Preosteoblast cells were differentiated by adding ascorbic acid and β-glycerophosphate, and co-treated with loganic acid (2, 10, and 50 μM). Bone formation is stimulated by either increasing proliferation of osteoblastic lineage or inducing osteoblast differentiation [30,31]. Our results showed that treatment with loganic acid did not affect the viability of MC3T3-E1 cells during the differentiation period (Figure 4A). However, loganic acid significantly increased ALP activity in a dose-dependent manner in ALP-positive cells (Figure 4B,C), indicating that loganic acid promoted osteoblast differentiation.

**Figure 5:**
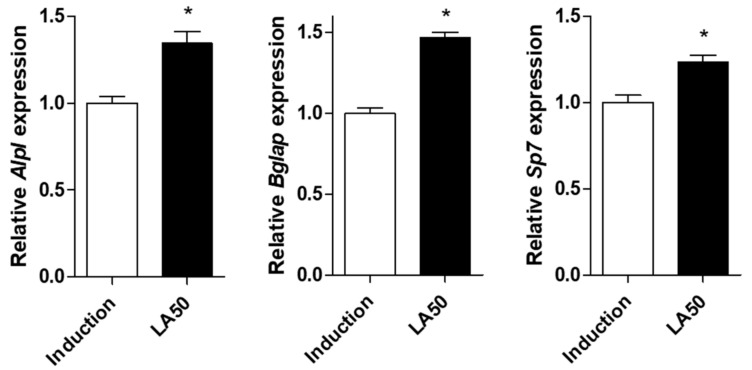
Effects of loganic acid on osteoblast differentiation-associated markers in MC3T3-E1 cells. After induction of osteoblast differentiation by co-treatment with 50 μM loganic acid, the mRNA expression levels of *Alpl*, *Bglap*, and *Sp7* were evaluated by qRT-PCR using targeted gene-specific primers, and the expressions were normalized using mouse *Gapdh* mRNA level. LA: loganic acid, Induction: osteoblast induction media-treated group. * *p* < 0.05 vs. Induction (Student’s *t*-test).

Next, we investigated the promotive effects of loganic acid on bone formation by assessing mRNA expression of bone-enhancing markers such as *Alpl* (alkaline phosphatase, ALP), *Bglap* (bone gamma-carboxyglutamic acid-producing protein, osteocalcin), and *Sp7* (osterix). In the present study, 50 μM loganic acid treatment increased mRNA expression levels of differentiation-associated markers of osteoblasts such as *Alpl*, *Bglap*, and *Sp7* (Figure 5). These results suggested that loganic acid induced osteoblast differentiation through upregulation of osteoblastogenesis-associated genes.

### 2.3. Loganic Acid Inhibited Primary-Cultured Osteoclast Cell Differentiation

**Figure 6:**
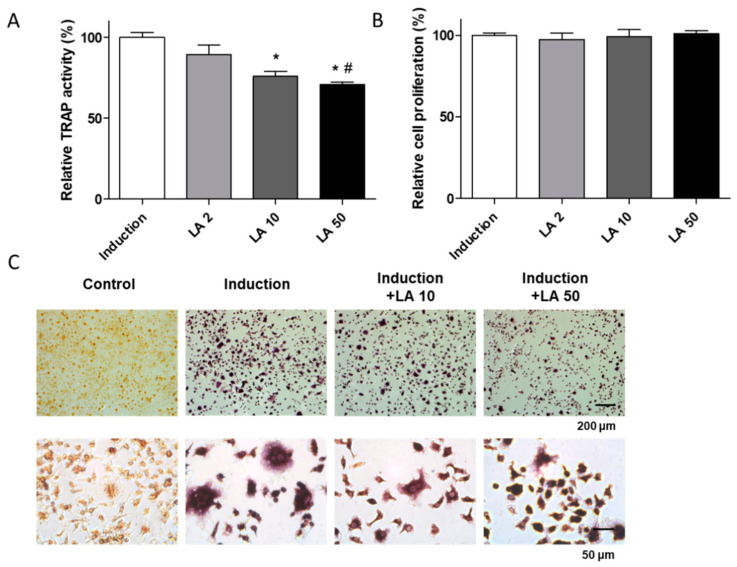
The effects of loganic acid on osteoclast differentiation. Primary-cultured monocytes from mouse femoral bone were co-incubated with osteoclast induction media (30 ng/mL of M-CSF and 50 ng/mL of RANKL) and different concentrations of loganic acid (2, 10, and 50 μM) for 5 days. (**A**) TRAP activity was assessed using an Acid-Phosphatase Kit. LA: loganic acid, Induction: osteoclast induction media-treated group. * *p* < 0.05 vs. Con, ^#^ *p* < 0.05 vs. LA2 (ANOVA with Tukey’s honest significant difference post hoc test). (**B**) Relative cell proliferation was evaluated by water-soluble tetrazolium (WST) assay. (**C**) TRAP-positive cells were stained and visualized by a light microscope.

To confirm the effects of loganic acid on bone resorption, we investigated the differentiation of monocytes (preosteoblastic cells) derived from bone marrow of a seven-week-old mouse. Monocytes were successfully isolated and confirmed using fluorescence-activated cell sorting analysis (Supplementary Figure S2). The isolated monocytes were differentiated into osteoclasts by adding the receptor activator of nuclear factor kappa-B ligand (RANKL) and monocyte-colony stimulating factor (M-CSF). After induction of osteoclast differentiation, cells were co-treated with loganic acid (2, 10, and 50 μM) and osteoclast differentiation was assessed by TRAP activity/staining. In the present study, loganic acid reduced TRAP activity of primary-cultured osteoclasts in a dose-dependent manner without affecting the cellular proliferation of monocytes (Figure 6A,B). In addition, stained TRAP-positive cells were decreased by treatment with loganic acid, compared to the induction group (Figure 6C). These results indicated that loganic acid inhibited osteoclast differentiation.

### 2.4. Oral Administration of Loganic Acid Prevented OVX-Induced Osteoporosis In Vivo

**Figure 7:**
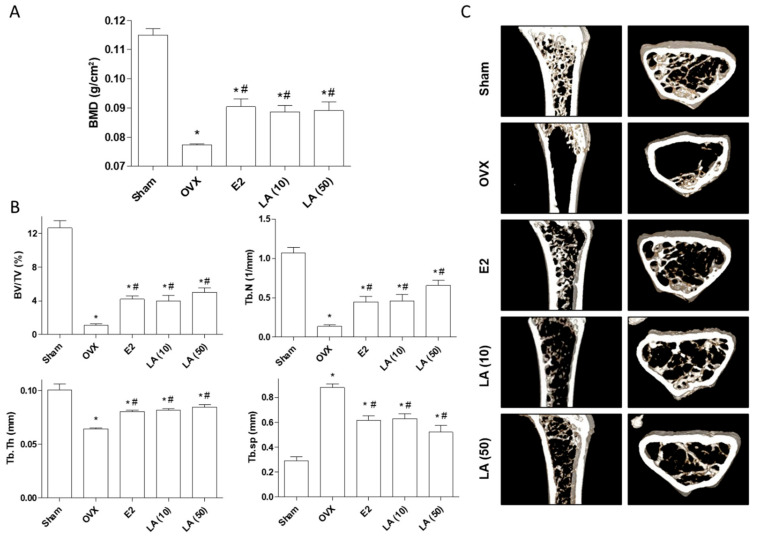
Effects of loganic acid on an OVX-induced osteoporosis murine model. Mice were administered either 17β-estradiol (E2) or loganic acid (10 or 50 mg/kg/day) for 12 weeks. Sham: sham operated, OVX: ovariectomized mice, LA: loganic acid. (**A**) Bone mineral density of the right femoral bone was evaluated by PIXI-mus bone densitometer after 12 weeks. (**B**) Bone volume (BV/TV), trabecular thickness (Tb.Th), number (Tb.N), and spacing (Tb.Sp) were measured after 12 weeks. (**C**) Micro-CT images of the right femoral bone were scanned by microcomputed tomography. * *p* < 0.05 vs. Sham, ^#^ *p* < 0.05 vs. OVX (ANOVA with Tukey’s honest significant difference post hoc test).

Based on the in vitro results, we further assessed the anti-osteoporotic effect of loganic acid in OVX-induced osteoporosis mice. Eight-week-old OVX mice were either orally administered with different concentrations of loganic acid (2, 10, and 50 mg/kg/day) or subcutaneously injected with 17β-estradiol (E2, 0.03 μg) (positive control group) for prevention of OVX-induced osteoporosis [32]. After 12 weeks of treatment, BMD of the right femoral bone was assessed using a PIXI-mus bone densitometer, and micro-CT bone structures were scanned for analysis of trabecular properties, such as bone volume (BV/TV), number (Tb.N), trabecular thickness (Tb.Th), and spacing (Tb.Sp). As expected, treatment of mice (positive control group) with E2 for 12 weeks inhibited OVX-induced BMD loss and rescued bone structural properties such as BV/TV, Tb.N, Tb.Th, and Tb.Sp when compared to the OVX group. Similarly, treatment with loganic acid prevented osteoporotic BMD loss in mouse femoral bone (Figure 7A). In addition, the quantitative parameters demonstrated that loganic acid enhanced protective effects against OVX-induced osteoporosis (Figure 7B). Micro-CT images of the right femoral bone revealed that loganic acid improved OVX-induced bone structural properties (Figure 7C). Taken together, these results suggested that loganic acid inhibited OVX-induced osteoporosis in vivo and can be considered as a therapeutic agent for the prevention of bone loss. 

## 3. Discussion

This study describes the antiosteoporotic effects of loganic acid derived from *Gentiana lutea* L. (GL) extract on osteoblastic and osteoclastic cells in vitro and in ovariectomized (OVX)-induced osteoporosis mice in vivo. Antiosteoprotective effects of GL extract were assessed through increased alkaline phosphatase (ALP) activity in osteoblast cells and reduced tartrate-resistant acid phosphatase (TRAP) activity in osteoclast cells. In the present study, we identified a bioactive compound, loganic acid, which was isolated from GL extract. Loganic acid enhanced the differentiation of osteoblast in preosteoblastic MC3T3-E1 cells but prevented the differentiation of primary-cultured osteoclast cells. Finally, loganic acid was found to exert protective effects against OVX-induced osteoporosis in mice.

GL extract comprises various bioactive components [28], and in this study, we showed that GL extract promotes bone formation using MC3T3-E1 preosteoblast cells. Loganic acid is one of the main compounds present in GL extract [28]. Several therapeutic effects of loganic acid have been demonstrated, including inhibitory effects on inflammation [33], anti-obesity effects [34], and preventive effects on diet-induced hypertriglyceridemia and atherosclerosis [35]. However, the protective effects of loganic acid on bone metabolic disorders have not been elucidated yet.

New bone formation is induced upon the differentiation of osteoblasts derived from mesenchymal stem cells, which is regulated via the transcription factors, such as ALP, osteocalcin, and osterix [36]. During osteoblast differentiation, an increase in the expression of ALP and osteocalcin regulates osteoblast function and bone mineralization [37,38]. Additionally, osterix is a zinc finger protein that is essential for osteoblast differentiation and bone formation [39]. Loganic acid increases ALP activity through increasing the expression of *Alpl*, *Bglap*, and *Sp7,* indicating that loganic acid stimulates osteoblast differentiation through activating osteoblast-inducing genes.

Bone remodeling is regulated via maintaining an equilibrium between osteoblasts (bone formation) and osteoclasts (bone resorption) [40]. Osteoporotic postmenopausal women with aggressive osteoclastogenesis are at a high risk of fragility fractures [41]. Osteoclasts are differentiated via the fusion of monocyte/macrophage lineage cells originating from hematopoietic stem cells [42]. TRAP is considered as a reliable biomarker for osteoclast differentiation associated with bone resorption and remodeling [43]. In the present study, loganic acid was found to rescue TRAP-positive cells and activity, suggesting that loganic acid inhibits osteoclast differentiation.

Ovariectomy involves the removal of ovaries and is a well-known postmenopausal model, resulting in estrogen deficiency, which plays an important role in bone homeostasis [44]. Dysregulation of bone homeostasis triggers the loss of bone mineral density (BMD), leading to osteoporosis in mice and humans [45]. OVX-induced mice exhibit significantly reduced BMD and abnormal trabecular bone structural properties. Loganic acid treatment inhibited osteoporotic BMD loss and improved quantitative bone structural properties, such as BV/TV, Tb.N, Tb.Th, and Tb.Sp. Moreover, micro-CT images revealed that loganic acid enhanced OVX-induced bone microarchitecture. These data suggest that loganic acid prevents osteoporotic trabecular bone loss in OVX mice.

However, there were several limitations in this study, such as it is still uncertain if loganic acid is the only component present in GL extract, which is responsible for the anti-osteoporotic effects. In this study, we examined OVX female mice; however, it is unclear whether the osteoprotective effects of loganic acid are sex-specific. Finally, detailed analyses of the anti-osteoporotic effects of loganic acid are required to be performed further, including the assessment of physiological bone metabolic status of bone formation and resorption in the femoral bone.

## 4. Materials and Methods

### 4.1. Fractionation, Isolation, and Structure Identification of Loganic Acid from Gentiana lutea L. Extract

GL root was dissolved in 30% ethanol for 24 h at room temperature and then dried under vacuum. The remaining aqueous solution was filtered, and then sequentially fractioned using chloroform, ethyl acetate (EtOAc), and butanol (BuOH) to obtain chloroform (102.1 mg), EtOAc (81.1 mg), and BuOH (452.7 mg) extracts, respectively. The anti-osteoporotic effects of each fraction were assessed by estimating ALP activity in MC3T3-E1 preosteoblast cells. The BuOH fraction was further fractionated by column chromatography using octadecylsilyl (ODS) gel column chromatography and then eluted using a 25% methanol solution into five subfractions (GL-B-01 to GL-B-05). Next, the GL-B-03 fraction was subjected to RP-18 column chromatography and eluted with an acetonitrile solution (containing 0.05% trifluoroacetic acid) in a step-gradient manner (10‒40%).

### 4.2. Cell Culture for Osteoblast, Osteoclast Differentiation

Mouse preosteoblast MC3T3-E1 cells (RIKEN Cell Bank, Tsukuba, Japan) were maintained in α-modified minimal essential medium (α-MEM; Gibco; Waltham, MA, USA) containing 10% fetal bovine serum (Gibco, Grand Island, NY, USA), penicillin (100 U/mL; Gibco, Grand Island, NY, USA), and streptomycin (100 μg/mL; Gibco, Grand Island, NY, USA). Preosteoblastic cells were differentiated by β-glycerophosphate (10 mM) (Sigma-Aldrich; St. Louis, MO, USA) and ascorbic acid (50 μg/mL) (Sigma-Aldrich; St. Louis, MO, USA) for 3 days. For osteoclast differentiation, bone marrow cells were harvested from the femur of a seven-week-old mouse, and the isolated monocytes were verified using a FACS Aria III cell sorter (BD Biosciences, San Jose, CA, USA) with the FACS Diva software (BD Biosciences; San Jose, CA, USA)). The monocytes were induced to be differentiated into osteoclasts by the addition of M-CSF (30 ng/mL; Peprotech, Rocky Hill, CT, USA) and RANKL (50 ng/mL; PeproTech; Cranbury, NJ, USA). All the cultured cells were grown at 37 °C in a cell culture incubator with 5% CO_2_.

### 4.3. Evaluation of ALP/TRAP Activity and Staining

For ALP activity, the differentiated osteoblasts were harvested and lysed with 1 mmol/L Tris-HCl pH 8.8 containing 0.5% Triton X-100 and 10 mmol/L Mg^2+^. The cell lysates were then incubated with 5 mmol/L p-nitrophenylphosphate (Sigma, St. Louis, MO, USA), and optical density was measured at an absorbance of 405 nm (Bio-Rad; Hercules, CA, USA). For ALP staining, the cells were fixed with 4% paraformaldehyde for 15 min and incubated with BCIP/NBT (Sigma-Aldrich; St. Louis, MO, USA) for 30 min at room temperature. To determine osteoclast differentiation, primary-cultured monocytes were lysed with cell lysis buffer, and TRAP activity/staining was performed using an Acid-Phosphatase Kit (Sigma, St. Louis, MO, USA), according to the manufacturer’s instructions. Images of ALP and TRAP-positive cells were obtained using a light microscope (KERN & SOHN GmbH; Balingen, Germany).

### 4.4. Water-Soluble Tetrazolium (WST) Assay

Cells were incubated in a 96-well plate overnight and co-treated with different concentrations of loganic acid (2, 10, and 50 μM). Cell viability was evaluated using the D-Plus™ CCK cell viability assay kit (Donginls; Seoul, Republic of Korea). WST reagent was added to each well and the plate was incubated for 4 h. Next, absorbance at 450 nm was measured using a microplate reader (BIO-RAD, Hercules, CA, USA).

### 4.5. Quantitative Reverse-Transcription Polymerase Chain Reaction (qRT-PCR)

Total RNA was isolated from cultured cells using the TRIzol reagent (Invitrogen, Carlsbad, CA, USA) according to the manufacturer’s instructions. RNA was reverse-transcribed into complementary DNA (cDNA) using the RevertAid™ H Minus First Strand cDNA Synthesis Kit (Fermentas, Hanover, NH, USA) with oligo (dT) 12–18 primers. PCR amplifications for osteoblastogenesis-associated genes were performed using the SYBR Green I qPCR Kit (TaKaRa, Shiga, Japan) and gene-specific primers. The specific primer sequences used in this study were as follows: forward 5′-CCA ACT CTT TTG TGC CAG AGA-3′ and reverse 5′-TGA CAT TCT TGG CTA CAT TGG TG-3′ for mouse *Alpl*; forward 5′-TAG TGA ACA GAC TCC GGC GCT A-3′ and reverse 5′-TGT AGG CGG TCT TCA AGC CAT-3′ for mouse *Bglap*; forward 5′-ATG GCG TCC TCT CTG CTT G-3′ and reverse 5′-TGT AGG CGG TCT TCA AGC CAT-3′ for mouse *Sp7*; and 5′-AGG TCG GTG TGA ACG GAT TTG-3′ and 5′-TGT AGA CCA TGT AGT TGA GGT CA-3′ for mouse *Gapdh*. By normalizing with mouse *Gapdh*, the relative gene expressions were determined as 2^−∆∆Ct^, and fold-changes were determined by comparing with the untreated induction group.

### 4.6. Ovariectomy (OVX)-Induced Osteoporosis Murine Model

Eight-week-old ovariectomized (*n* = 20) or sham-operated (*n* = 5) ddY mice were purchased from Shizuoka Laboratory Center, Inc. (Hamamatsu, Japan). The mice were provided with sterile food pellets (3.0–5.0 g/day, Feedlab Co., Ltd., Hanam, Korea) and autoclaved water (15 mL/day). All the mice were housed in filter-top cages and maintained under controlled conditions: temperature (23 ± 2 °C), humidity (55 ± 5%), and illumination (12-h light/12-h dark cycle). For the experiment, the mice were administered different concentrations of loganic acid (2, 10, and 50 mg/kg/day) by oral gavage or 17β-estradiol (E2, 0.03 μg, Sigma, St. Louis, MO, USA) by subcutaneous injection for 12 weeks. All the animal experiments were approved by the Institutional Animal Care and Use Committee (IACUC) of Ajou University School of Medicine (AMC-133; 1 January 2017) and conducted according to the institutional guidelines of the committee.

### 4.7. Examination of Bone Marrow Density (BMD) and Micro-CT Imaging in Mice

Mice were anesthetized by intraperitoneal injection of tiletamine/zolazepam (Zoletil, Virbac Laboratories, Carros, France), and BMD of the right femur was measured using a PIXI-mus bone densitometer with on-board PIXI-mus software (GE Lunar, Madison, WI, USA). For micro-CT imaging, the right femoral bone was dissected and scanned by Inveon micro-CT (INVEON, SIEMENS, Munich, Germany) in Gyeonggido Business & Science Accelerator (GBSA, Suwon, Korea). Qualitative analysis of two-dimensional axial and 3D images was performed using CTvox software (BRUKER, Billerica, MA, USA).

### 4.8. Statistical Analysis

Data in the bar graphs are presented as mean ± standard error of the mean (SEM). All statistical analyses were performed using GraphPad Prism 6.0 software (GraphPad Software, San Diego, CA, USA). Statistical significance between two groups was calculated by Student’s *t*-test. Comparison of multiple groups was performed by one-way analysis of variance (ANOVA) with Tukey’s honest significant difference (HSD) post hoc test. A probability value (*p*) less than 0.05 was considered statistically significant.

## 5. Conclusions

In this study, we investigated osteoprotective effects of the phytochemical compound, loganic acid, isolated from GL extract. Loganic acid promoted osteoblast differentiation through upregulation of osteoblastogenesis-associated genes (*Alpl*, *Bglap*, and *Sp7*) and inhibited osteoclast differentiation. In vivo experiments performed in an osteoporotic mice model demonstrated that loganic acid treatment prevented OVX-induced bone loss and improved trabecular bone structure properties. Taken together, our results suggest that loganic acid may be a potential candidate to be used as an anti-osteoporotic agent.

## Supplementary Materials

The following are available online at https://www.mdpi.com/1422-0067/22/1/233/s1.
