# Supplementary material for: Osteoprotective Effects of Loganic Acid on Osteoblastic and Osteoclastic Cells and Osteoporosis-Induced Mice
Source: Int J Mol Sci. 2020 Dec 28;22(1):233. doi: 10.3390/ijms22010233 (PMC7795511; doi:10.3390/ijms22010233)
Supplement: Supplementary file 1 [file ijms-22-00233-s001.pdf]

Osteoprotective Effects of Loganic Acid on Differentiation of Osteoblast and Osteoclast, and Ovariectomized Mice

Eunkuk Park, Chang Gun Lee, Eunguk Lim, Seokjin Hwang, Seung Hee Yun, Jeonghyun Kim, Hyesoo Jeong, Yoonjoong Yong, Seong-Hoon Yun, Chun Whan Choi, Hyun-Seok Jin and Seon-Yong Jeong

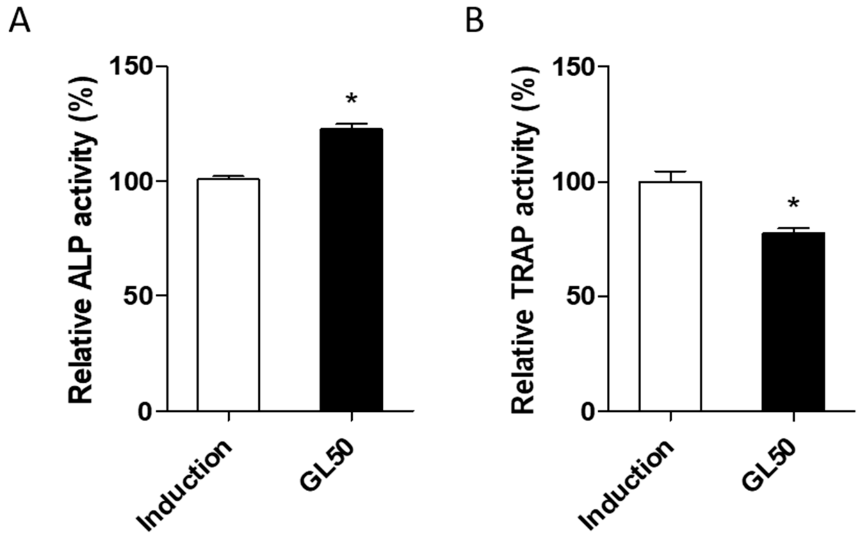

Supplementary Figure S1. Effects of a 30% ethanol extract of *Gentiana lutea* (GL) root on osteoblast and osteoclast differentiation. (A) MC3T3-E1 cells were co-incubated with osteoblast induction media (induced with ascorbic acid and β-glycerophosphate) and GL root 30% ethanol extract (GL50; 50 μg/mL) for 3 days. Osteoblast differentiation was assessed by ALP activity. (B) Mouse primary monocytes were co-incubated with osteoclast induction media (Induced with M-CSF and RANKL) and 30% ethanol extract of GL root (GL50; 50 μg/mL) for 3 days. Osteoclast differentiation was determined by TRAP activity. *$p < 0.05$ vs. Induction (Student's t-test).

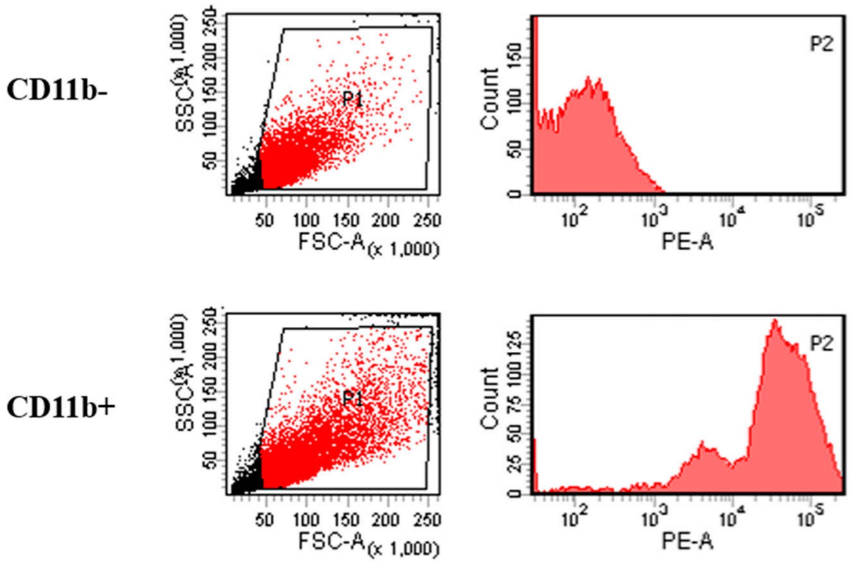

Supplementary Figure S2. Identification of monocytes isolated from mouse femoral bone marrow. Primary-cultured monocytes were identified by fluorescence-activated cell sorting analysis using a monocyte-specific surface marker (phycoerythrin-conjugated CD11b antibody).
